# Biomarkers of Response to Neoadjuvant Androgen Deprivation in Localised Prostate Cancer

**DOI:** 10.3390/cancers14010166

**Published:** 2021-12-29

**Authors:** Maree Pechlivanis, Bethany K. Campbell, Christopher M. Hovens, Niall M. Corcoran

**Affiliations:** 1 Department of Surgery, University of Melbourne, Parkville, VIC 3050, Australia; bethany.campbell@unimelb.edu.au (B.K.C.); chovens@unimelb.edu.au (C.M.H.); con@unimelb.edu.au (N.M.C.); 2 Department of Urology, Royal Melbourne Hospital, Parkville, VIC 3050, Australia; 3 Department of Urology, Western Health, Footscray, VIC 3011, Australia; 4 Victorian Comprehensive Cancer Centre, Melbourne, VIC 3000, Australia

**Keywords:** prostate cancer, neoadjuvant, androgen deprivation therapy, resistance

## Abstract

**Simple Summary:**

Prostate cancer is the second leading cause of cancer deaths in men. Attempts to improve patient outcomes include trials of neoadjuvant androgen deprivation therapy for patients with high-risk disease. Neoadjuvant treatment refers to androgen deprivation therapy that is administered prior to surgery (or radiation therapy). Patients typically respond well to this treatment regimen, showing a decrease in tumour size, but a significant proportion of patients eventually relapse and progress to metastatic disease. The mechanisms driving this resistance to neoadjuvant treatment are currently unknown. This review explores theories of resistance broadly, and their possible applications in the prostate cancer setting. Additionally, this review draws comparisons between breakthrough resistance and neoadjuvant resistance, and lastly investigates the current biomarkers for treatment sensitivity.

**Abstract:**

Prostate cancer (PCa) is a hormone driven cancer, characterised by defects in androgen receptor signalling which drive the disease process. As such, androgen targeted therapies have been the mainstay for PCa treatment for over 70 years. High-risk PCa presents unique therapeutic challenges, namely in minimising the primary tumour, and eliminating any undetected micro metastases. Trials of neoadjuvant androgen deprivation therapy aim to address these challenges. Patients typically respond well to neoadjuvant treatment, showing regression of the primary tumour and negative surgical margins at the time of resection, however the majority of patients relapse and progress to metastatic disease. The mechanisms affording this resistance are largely unknown. This commentary attempts to explore theories of resistance more broadly, namely, clonal evolution, cancer stem cells, cell persistence, and drug tolerance. Moreover, it aims to explore the application of these theories in the PCa setting. This commentary also highlights the distinction between castration resistant PCa, and neoadjuvant resistant disease, and identifies the markers and characteristics of neoadjuvant resistant disease presented by current literature.

## 1. Introduction

Prostate epithelial cells rely on androgens for growth and survival, and the androgen signalling axis plays a pivotal role in the pathogenesis and progression of prostate cancer (PCa) [1,2]. As such, interfering with androgen receptor signalling has been the cornerstone of treatment approaches to advanced disease for over 70 years [1]. Broadly speaking, these androgen deprivation therapies (ADT) disrupt the androgen receptor (AR) signalling axis either by inhibiting androgen biosynthesis, or inhibiting normal AR function.

PCa can be stratified into low, intermediate, and high-risk categories, which are associated with an increasing risk of failure of local treatments, as well as the development of metastases and death. These categories are assigned by considering tumour invasiveness, serum prostate specific antigen (PSA) levels and tumor grade (the Gleason score) [3]. High-risk disease denotes a locally advanced tumour with PSA > 20 ng/mL and a Gleason score of 8–10 [3]. Two therapeutic challenges arise when treating patients with high-risk PCa: firstly, controlling the primary tumour to prevent growth, and secondly, halting the invasion of adjacent tissues and achieving systemic control of micro metastases. This necessitates a multimodal treatment approach and may include neoadjuvant androgen deprivation therapy (nADT), which attempts to address both challenges [4]. nADT refers to administering ADT prior to surgery (or radiation) instead of after [4]. Clinical outcomes differ for nADT given prior to surgery compared to radiation therapy. When used in combination with external beam radiotherapy, ADT (neo-adjuvant and adjuvant) improves long term oncological outcomes in high and intermediate risk disease, which is not to the case with surgery [5]. For the purpose of this commentary, however, nADT refers to ADT given prior to radical prostatectomy, as individual mechanisms of treatment resistance and identification of specific biomarkers of response are harder to deconvolve when two modalities that affect cell survival are given concomitantly. In surgical patients nADT may be used to reduce the size of locally advanced tumours, facilitating surgical resection. Patients undergoing nADT typically show a reduction in tumour size at the time of surgery, but most patients still develop resistance and the PCa progresses to metastatic disease. Presently, nADT is only administered within the context of clinical trials as this treatment regimen has been unable to produce significant improvements in overall survival (OS) [4].

**Figure 1:**
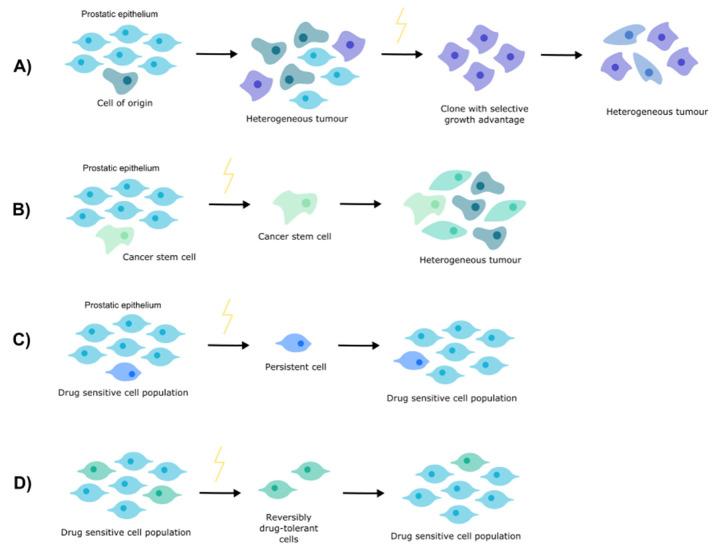
Theories of resistance. (**A**) Clonal evolution. (**B**) Cancer stem cells. (**C**) Cell persistence. (**D**) Drug tolerance.

Understanding how PCa develops resistance to nADT necessitates an appreciation of resistance more broadly. There are a number of theories concerning tumorigenesis and treatment resistance (Figure 1).

## 2. Clonal Evolution

In the 1970s, Nowell proposed a model for tumour evolution, whereby a single cell gives rise to mutant clones that expand and mutate in parallel. This process is termed clonal evolution [6].

Nowell posited that neoplasia begins with the expansion of a single cell. This ‘cell of origin’ has escaped normal growth controls and possesses a selective growth advantage over the parent cell population. The genetic instability of this cell predisposes it to producing mutant daughter cells upon replication. If a daughter cell acquires an additional selective growth advantage it will persist and give rise to a subpopulation within the tumour [6]. If different cells display similar growth advantages simultaneously, multiple subpopulations can coexist [7]. The result is a heterogeneous tumour, constantly evolving and consisting of multiple different sub clonal populations at any given point in time [8].

The dynamics of clonal evolution are determined by the mutation and expansion rates of different clonal subpopulations. Mutation rates are variable across distinct genomic regions, mutation types [9], and for epigenetic changes [7]. In response to environmental pressures, natural selection acts on tumours, positively selecting for specific subclones that express favourable genetic or epigenetic changes [6,8,10].

Pharmacological intervention is an example of environmental pressure. Whilst these interventions might eliminate certain clonal subpopulations, they could also serve as positive selective pressures for inherently resistant clones [8]. For instance, clones that possess mutated drug targets or constitutive signalling have a growth advantage over other subpopulations and will thrive in the presence of treatment. An example which highlights the implications of clonal evolution for treatment outcomes is PCa, where clones harbouring mutations in the AR or components of the AR signalling axis are able to resist ADT [11].

## 3. Cancer Stem Cells

A second model for tumorigenesis, the cancer stem cell (CSC) model, postulates that tumours consist of a subset of cells known as CSCs which are the driving force for tumour development, progression, and resistance [12]. CSCs also supposedly contribute to tumour heterogeneity as they can self-renew and differentiate, giving rise to diverse tumour cell populations, some of which may metastasise or develop resistance [13].

CSCs share similarities with normal stem cells, namely, multipotency and their ability to self-renew [14,15]. They are a dynamic cell type, with variations in CSC phenotype occurring across different cancer subtypes and even within the same cancer over the life of the tumour [16,17]. There are also differences between normal stem cells and CSCs. Whilst growth and differentiation are strictly regulated in normal stem cells, mutations cause CSCs to exhibit uncontrolled growth and differentiation [13,18]. Unlike normal stem cells, CSCs are also tumorigenic, meaning they can form tumours when transplanted into animals [19].

CSCs often have a number of characteristics that afford resistance to treatment, including acquired proliferative dormancy, drug efflux mechanisms, enhanced DNA repair, decreased apoptosis, and favourable interactions with their microenvironment [16]. In physiological contexts, stem cells dormancy is necessary for tissue homeostasis; in pathological contexts, dormancy allows CSCs to evade chemotherapeutics that target rapidly proliferating cells [20]. CSCs can express multi drug resistance transporters which efflux cytotoxic drugs [21]. CSCs also repair DNA damage more efficiently than normal cells, affording them resistance to radiation therapy (RT) [22]. Additionally, CSCs upregulate reactive oxygen species (ROS) scavengers, protecting them from ROS-mediated DNA damage, a mechanism by which RT causes cell death [23]. Apoptosis is also altered in CSCs due to dysregulation of key apoptotic pathway proteins [24]. For example, the mitochondrial Bcl-2 family of proteins are upregulated in a number of cancers, including PCa, where it is associated with resistance to the chemotherapeutic, docetaxel [25]. Like normal stem cells, CSCs rely on their microenvironment, the stem cell niche, to retain their self-renewal capabilities and remain undifferentiated [26]. Components of the stem cell niche include stromal cells, inflammatory cells, and blood vessels which interact with and support CSCs in maintaining their phenotype, enabling the CSCs to enact resistance to treatment and support tumour survival [27].

## 4. Cell Persistence

In both cancer and infectious disease management, analogous challenges are faced, namely in identifying the mechanisms by which cells persist after treatment and developing therapeutics that can eliminate these persistent cells. Parallels can be drawn from microbiology research to facilitate understanding of cancer cell resistance.

In the context of infectious disease, ‘persister cells’ are cells which evade treatment by antibiotics but are distinct from antibiotic resistant cells. Unlike resistant cells, persister cells lack any drug-resistance mutations [28]. Additionally, persister cells are sensitive to antibiotic treatment, but do not proliferate during treatment. This allows them to resist treatment that targets proliferating cells. Once treatment is withdrawn these cells will spontaneously resume proliferation, giving rise to a population of cells that are equally as sensitive as the original persister to the bactericidal agent [29,30].

The phenomenon of bacterial persistence shares similarities with CSC-mediated relapse. In CSC-mediated resistance, treatment is initially effective and the tumour regresses, but a small population of cancer cells persist and initiate relapse [28]. Like bacterial persisters, these residual cancer cells lack any resistance mutations that could explain their persistence in the presence of treatment. Moreover, when these residual cancer cells are expanded, they give rise to a population of cancer cells that are drug sensitive. It is hypothesized that these persistent cells are CSCs as they express cell-surface antigens consistent with those found on tissue stem cells [28]. If this hypothesis is correct, then it can be assumed that the CSCs employ the various mechanisms outlined earlier such as proliferative dormancy, enhanced DNA repair, and decreased apoptosis to resist treatment.

## 5. Drug Tolerance

A phenomenon known as re-treatment response is being increasingly observed in cancer [31]. It describes the scenario in which a drug is administered, and a response is observed but is eventually followed by treatment failure. Treatment is then withdrawn and following a ‘drug holiday’ the same drug is re-administered, and the patient displays a second treatment response [32,33]. This suggests that initial resistance to treatment is due to a reversible drug-tolerant state [31].

Sharma et al. investigated this drug-tolerant state in the PC9 pulmonary adenocarcinoma cell line [31]. They found that following high-dose drug treatment a small population of cells that were drug-tolerant persisted. These cells, termed drug-tolerant persisters (DTPs), were non-proliferative and expressed stem-cell markers. In drug treatment conditions approximately 20% of the DTPs underwent a phenotype change, losing their stem-cell markers and resuming normal proliferation. These proliferative cells were termed drug-tolerant expanded persisters (DTEPs). It was also shown that treatment-free passaging of both DTPs and DTEPs resensitised the cells to treatment, highlighting the reversible nature of the drug-tolerant state. They concluded that the in vitro cancer cell population consists of three discrete subpopulations: the parental PC9 cells, DTPs, and DTEPs. These three cell types were genetically identical, yet displayed differing functional phenotypes, suggesting a role of the epigenome in developing and regulating the reversibly drug-tolerant state. Further analyses identified a crucial role of the chromatin state in sustaining drug-tolerant cell populations. KDM5A, a histone demethylase, was recognised as a crucial chromatin-modifying enzyme in the establishment of the drug-tolerant state [31]. A role for KDM5A in drug resistance has also been suggested by others [34,35] and was investigated by Vinogradova et al. who showed that KDM5A messenger ribonucleic acid (mRNA) was increased in drug-tolerant cell models. They also showed that combination therapy with a KDM5A inhibitor (CPI-455) decreased the number of cells that could survive a lethal drug dose in multiple cell culture models [36]. More recently it has been recognized that these epigenetic changes drive loss of epithelial markers with only partial acquisition of mesenchymal markers in drug tolerant cells [37,38]. This drug tolerant plasticity, characterized by an incomplete EMT switch, appears important in acute stress survival, contributing to cell persistence during drug challenges. These findings support the hypothesis that reversible drug tolerance is epigenetically moderated.

The transiently drug-tolerant state of these cells is also analogous to bacterial persister cells. Whilst some studies equate bacterial persister cells with CSCs [28], Sharma et al. contends that bacterial persisters are instead homologous with DTPs, and that DTPs are distinct from CSCs. This is based on the observation that both bacterial persisters and DTPs exhibit phenotypic heterogeneity which is not genetically regulated. When treatment is ceased, both DTPs and bacterial persisters give rise to subpopulations that are as drug-sensitive as the original persistent cells [31]. Sharma et al. also suggests that DTPs are distinct from CSCs as they do not employ drug efflux mechanisms to evade treatment. Presently a clear consensus does not exist, but current literature indicates a likely interconnection between the CSC and the reversibly drug-tolerant cell populations [31,39,40,41].

## 6. Castration Resistance

Castration resistant prostate cancer (CRPC) refers to cancer which progresses in the androgen-depleted environment created by ADT [42,43]. In the majority of patients, PCa is initially hormone-sensitive and patients respond well to ADT, showing significant tumour regression, a fall in serum prostate specific antigen levels, and improvements in quality of life [44]. However, some cancer cells persist in the androgen-depleted environment, resulting in androgen-independent, or castration-resistant cancer [42,45].

A number of mechanisms of castration resistance have been identified (Table 1), including amplification and/or mutation of the AR, constitutively active AR splice variants, increased intracrine androgen synthesis, altered expression or activity of AR coactivators or corepressors, and increased androgen biosynthesis by conversion of non-testicular androgens in peripheral tissue to potent androgens [46,47]. These mechanisms enable sustained signalling through the AR in the androgen-depleted environment.

A second hypothesis detailing the mechanism of CRPC suggests that a subpopulation of neuroendocrine (NE) cells within the cancer cell population act as autocrine-paracrine signallers to encourage a more aggressive cancer phenotype [48,49,50]. These NE cells originally arise from adenocarcinoma and differentiate, producing secretory products which promote tumour cell proliferation, prevent apoptosis, and encourage angiogenesis in the androgen-depleted environment [51]. NE PCa has low or no expression of the AR and androgen-regulated genes (ARGs). This impacts treatment options, specifically the efficacy of ADT [48,51,52]. The NE differentiation is more prominent following ADT [51] and represents a possible mechanism by which previously responsive, hormone-sensitive cancer develops resistance to ADT [45,48,53].

## 7. Neoadjuvant Androgen Deprivation Therapy Resistance

Patients treated with nADT typically show a reduction in tumour volume and significant decreases in extra-prostatic disease [54,55,56,57] but complete pathological responses are rare and ultimately there is little to no improvement in OS [4]. Clinical trials assessing the effects of nADT have shown that this treatment regimen can produce appreciable improvements in the burden of local disease at the time of surgery. Importantly, ADT has been shown to decrease the rates of extracapsular extension, which describes the presence of neoplastic cells in the periprostatic tissue. ADT also increases negative surgical margins and lowers the frequency of lymph node metastases after radical prostatectomy (RP). Despite these favourable outcomes, trials of nADT have been unable to show a translation of these positive effects to improvements in progression-free survival or OS [54,55,56,57,58,59,60,61,62,63,64,65,66].

Identification of the characteristics of nADT resistant disease, specifically those differentiating it from CRPC (Table 1), are of significance as they serve as potential biomarkers for treatment response.

Wang et al. suggested that a greater insight into predictive morphological parameters and molecular markers of nADT-resistant tumours could aid in identifying subsets of patients likely to develop resistance to nADT. Core needle biopsies revealed that tumour cribriform growth pattern, macro-nucleoli, ductal adenocarcinoma differentiation, and *PTEN* loss in the untreated tumour were associated with treatment resistance [66]. This suggests that patients possessing these cellular and morphological characteristics may be unsuitable candidates for nADT.

Unlike CRPC, the mechanisms causing patient relapse and driving resistance to treatment in the neoadjuvant setting are yet to be determined, representing a gap in current literature and a possible therapeutic target for combination therapy.

Whilst the mechanisms of nADT resistance are not clearly defined, a number of characteristics of resistant disease have been identified. McKay et al. examined outcomes of a randomized phase II trial of neoadjuvant enzalutamide (a second generation antiandrogen) or leuprolide (a gonadotropin releasing hormone agonist), with or without abiraterone (an inhibitor of androgen biosynthesis). They showed that *ERG*-positive and *PTEN*-loss tumours harboured greater residual disease. Interestingly, *ERG*-positive, and *PTEN*-loss tumours were associated with reduced AR expression, suggesting androgen-independent survival mechanisms in these tumours. These findings also suggest an inherent insensitivity to ADT which could explain the extensive residual tumours observed in these patients [67].

Sowalsky et al. examined residual PCa foci in RP samples from patients treated with nADT. Transcriptome profiling of residual tumours revealed that AR signalling was reduced but continual—a commonality between nADT-resistant and castration-resistant disease [68]. Whole exome sequencing (WES) revealed a genomic loss in the tumour suppressor gene *RB1*. Moreover, a significant negative correlation was found between *RB1* mRNA levels and proliferation index, which suggests that downregulation of *RB1* could be driving proliferation. In cases where multiple tumour foci were micro dissected, WES revealed that the foci shared a common clonal origin; however, each focus had multiple unique oncogenic alterations. This suggests that a subpopulation of cells harbouring oncogenic alterations commonly observed in CRPC were present within the primary tumour, and were positively selected for by nADT [69].

Sowalsky et al. highlighted several distinctions between CRPC and nADT-resistant PCa. *RB1* loss is typically associated with NE differentiation in CRPC; however, Sowalsky et al. did not find a significant correlation between *RB1* loss and NE markers such as chromogranin A, chromogranin B, or synaptophysin in residual tumours. AR activity in residual tumour foci was not positively correlated with aldo-keto reductase family 1 member c3 expression, an enzyme-encoding gene whose expression drives intratumoral androgen synthesis in CRPC. Additionally, no increase in the expression of *AR* splice variant *AR-V7* was observed in neoadjuvant resistant tumour foci, contrary to CRPC where increased *AR-V7* is correlated with resistance to ADT [69,70].

## 8. Biomarkers of Resistance

For the first time, Cmero et al. [71] identified loss of *SNAI2* as a marker for AR sensitivity. Specifically, *SNAI2* deficient tumour cells display increased sensitivity to AR signalling inhibition.

Snai2 is a member of the Snail superfamily which are transcription factors implicated in EMT. Snail family members modulate EMT by binding the gene promoters of epithelial factors to repress their expression [72,73]. They also simultaneously promote the expression of mesenchymal factors [74,75] and other EMT-promoting transcription factors [46,75,76]. Upregulation of mesenchymal markers promotes migratory and invasive characteristics in the epithelial cells and causes significant phenotypic alterations [77]. Aberrant expression of members of the Snail superfamily has a myriad of pathological consequences. Their expression can promote changes in cell morphology, loss of normal cell–cell contacts, acquisition of invasive growth properties, and resistance to DNA-damage-indued cell death [78].

In the PCa setting, in vitro studies have shown that Snai2 overexpression is associated with increased proliferation, invasiveness [79,80], and resistance to treatments such as chemotherapy [81,82], radiation therapy [83,84], and tyrosine kinase inhibitors [85,86]. Additionally, Cmero et al. showed that Snai2 expression is upregulated in patients receiving acute ADT [71], and moreover, upregulation of Snai2 is associated with poor prognosis in a number of cancer contexts [87,88,89,90]. Given that Snai2 is important in the cell plasticity associated with ‘drug-tolerant persister’ cells, this suggests that loss of this plasticity renders prostate epithelial cells lethally vulnerable to the stress associated with acute androgen receptor signalling inhibition.

## 9. Combination Therapy to Overcome Resistance

Drug resistance is a major barrier to cancer treatment. One strategy that has been employed to overcome resistance is combination therapy. Accumulating evidence over the last 5 years indicates that upfront combination therapies in metastatic hormone response prostate cancer delays the onset of castration-resistance and results in greater gains in overall survival compared to traditional treatment sequencing. Agents successfully used in combination include docetaxel, a taxane based chemotherapy which because of its distinct mechanism of cytotoxic action synergises with ADT to maximise cell kill [91,92]. However, similar survival gains have been seen with novel androgen receptor signalling inhibitors such as abiraterone [93] and enzamlutamide [94], presumably as conventional ADT does not result in full AR signalling axis suppression. In the neo-adjvuant setting use of these agents also results in improved response rates [95,96] but whether this translates into improved overall survival is as yet unclear, with a number of phase III studies underway. A complementary approach is to combine ADT with agents that might disrupt pathways that are important in prostate cancer survival. Examples include ADT in combination with IMC-A12 (a monoclonal antibody against IGF-1R, trial ID: NCT00769795); ipatasertib (a small molecule AKT inhibitor; trial ID: NCT04737109), and erdafitinib (a tyrosine kinase inhibitor of FGFR1-4, trial ID: ACTRN12618001061224). The results of these studies are awaited.

## 10. Conclusions

The molecular mechanisms driving the persistence of prostate tumour cells during acute AR signalling inhibition appear distinct from those responsible for breakthrough, progressive castration resistant disease. Initial evidence suggests that tumour cells with low AR signalling dependence may be inherently resistant, while cells lacking the machinery required for an adaptive acute stress response are intrinsically sensitive. As such an increasing number of molecular markers are associated with treatment response, but these require larger validation studies to assess utility in the clinical setting.

## Figures and Tables

**Table 1:** Summary of the Unique Mechanisms and Characteristics of Disease.

Castration Resistant Prostate Cancer	Neoadjuvant ADT Resistant Prostate Cancer
Amplification and/or mutation of the AR	Cribriform growth pattern
Constitutively active AR splice variants	Macro-nucleoli
Increased intracrine androgen synthesis	Ductal adenocarcinoma differentiation
Altered expression or activity of AR coactivators or corepressors	PTEN loss
Increased androgen biosynthesis	ERG-positive and PTEN loss
Neuroendocrine differentiation	RB1 loss
